# A nomogram including body composition parameters for predicting recurrence of pT1 clear cell renal cell carcinoma: a multicenter retrospective study

**DOI:** 10.1186/s13244-025-02202-3

**Published:** 2026-02-02

**Authors:** Haonan Chen, Lingkai Cai, Juntao Zhuang, Yiran Tao, Zhengye Tan, Hao Yu, Chang Chen, Qikai Wu, Qiang Cao, Bo Liang, Pengchao Li, Xiao Yang, Qiang Lu

**Affiliations:** 1 Department of Urology, The First Affiliated Hospital of Nanjing Medical University, Nanjing, China; 2 Department of Urology, Wuxi Medical Center of Nanjing Medical University, Wuxi, China; 3 Department of Radiology, Union Hospital, Tongji Medical College, Huazhong University of Science and Technology, Wuhan, China; 4 Department of Urology, The Affiliated Suqian First People’s Hospital of Nanjing Medical University, Suqian, China

**Keywords:** Body composition parameters, Clear cell renal cell carcinoma, Computed tomography, Nomogram, Recurrence

## Abstract

**Objective:**

To develop and validate a body composition parameters (BCPs)-based nomogram for predicting recurrence in T1-stage clear cell renal cell carcinoma (ccRCC), comparing its performance with established models while exploring potential biological mechanisms.

**Materials and methods:**

536 patients from three institutions (training cohort: 343, external validation cohort: 193) were included. Univariate and multivariate Cox regression analyses identified independent prognostic factors for recurrence-free survival (RFS), which were incorporated into the nomogram. The model performance was evaluated, and potential biological mechanisms were explored.

**Results:**

The postoperative nomogram included three independent adverse prognostic factors for RFS: high Leibovich score (HR = 2.18, 95% CI: 1.44–3.31), high visceral adipose tissue density (VATD; HR = 2.34, 95% CI: 1.33–4.12), and high intramuscular adipose tissue content (IMAC; HR = 3.60, 95% CI: 1.29–10.07). The nomogram demonstrated superior discrimination, with a C-index of 0.732 (95% CI: 0.655–0.810) in the training cohort and 0.766 (95% CI: 0.677–0.855) in the validation cohort. The area under the curves (AUCs) for predicting 3- and 5-year RFS were 0.761 and 0.709 (training), and 0.844 and 0.765 (validation), outperforming the TNM, Leibovich, and SSIGN models. Through 5-fold cross-validation within the training cohort, the model achieved mean AUCs of 0.761 (3-year) and 0.683 (5-year). Calibration curves showed good consistency. Decision curve analysis indicated favorable clinical utility. Risk stratification (cutoff = 94.18) based on nomogram scores revealed significant RFS differences. Exploratory in silico analyses of transcriptomic data suggested enrichment in distinct cancer-related and metabolic pathways, as well as varying drug sensitivities between cohorts.

**Conclusion:**

The BCPs-based nomogram effectively predicts recurrence of T1 ccRCC and significantly improves upon existing prognostic models.

**Critical relevance statement:**

The nomogram, combining body composition parameters and Leibovich score, outperformed established prognostic models in predicting T1 ccRCC recurrence, enabling personalized risk stratification.

**Key Points:**

- Body composition parameters correlate with oncological outcomes in RCC, but remain underexplored in the T1 clear cell subtype.
- Elevated Leibovich score, visceral adipose tissue density, and intramuscular adipose tissue content independently predicted reduced RFS, linked to cancer-related and metabolic pathways enrichment.
- The body composition parameters-based nomogram could effectively predict postoperative recurrence in T1 ccRCC patients.

**Graphical Abstract:**

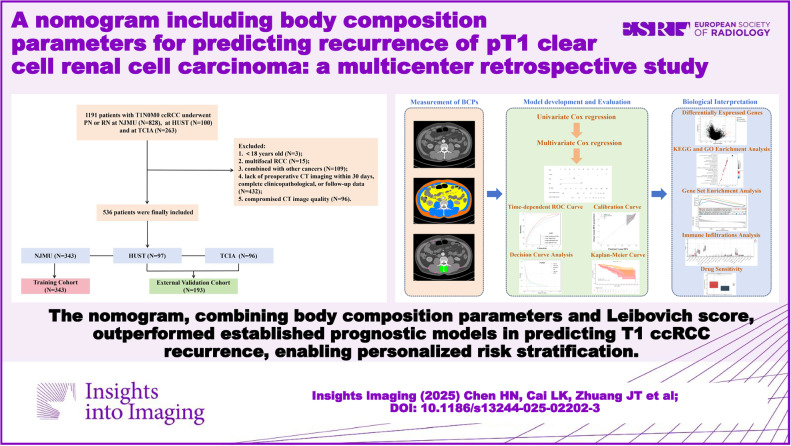

## Introduction

Currently, the widespread adoption of abdominal imaging has significantly enhanced early detection of localized renal cell carcinoma (RCC), and stage T1 tumors were also predominant in most studies [1]. Additionally, T1 clear cell RCC (ccRCC), representing the most prevalent histologic subtype, is characterized by a favorable prognosis [2], with a low postoperative recurrence rate (generally reported to be ≤ 10%) [3]. However, this excellent survival obscures a critical clinical challenge: the substantial number of patients requiring long-term surveillance to capture a relatively small number of recurrence events, leading to significant follow-up burden and healthcare costs. Seminal work by Sorokin et al [3] demonstrated that recurrences are common beyond 3 years after surgery, underscoring the need for extended, and potentially inefficient, monitoring protocols. Therefore, this prognostic uncertainty emphasizes the critical need for refined predictive models to optimize personalized follow-up strategies and postoperative risk stratification. Existing prognostic models, such as the 2003 Leibovich score [4], tumor-node-metastasis (TNM) system, and the stage, size, grade and necrosis (SSIGN) score [5], provide a foundation for risk assessment but have limitations in precisely stratifying T1 ccRCC patients, and their performance has considerable room for improvement.

Body composition parameters (BCPs) derived from CT imaging, which reflect the quantity and quality of region-specific muscle and adipose tissue, have emerged as novel prognostic biomarkers in oncology, demonstrating significant associations with clinical outcomes across multiple malignancies such as colorectal cancer, lung cancer, and hepatocellular carcinoma [6–8]. Nevertheless, prognostic correlations of BCPs vary across RCC subtypes, likely attributable to the high tumor heterogeneity and pathological distinctions: skeletal muscle parameters correlating with overall mortality in metastatic RCC [9]; subcutaneous/perirenal adipose tissue influencing localized papillary RCC progression [10]; and high visceral adipose radiodensity associating with poor overall and disease-free survival of ccRCC, potentially due to the role of lipid-depleted visceral fat (indicated by increased radiodensity) in promoting tumor progression through fatty acid transfer [11]. Notably, systematic investigation of the T1 ccRCC subtype reveals a critical knowledge gap in prognostic research, and the clinical value of BCPs in this population remains under-investigated.

Consequently, this study aimed to develop and validate a prognostic prediction model incorporating BCPs for predicting recurrence in T1 ccRCC patients, and to compare its performance against established models to assess its incremental value and clinical applicability.

## Patients and methods

### Study design and population

**Fig. 1:**
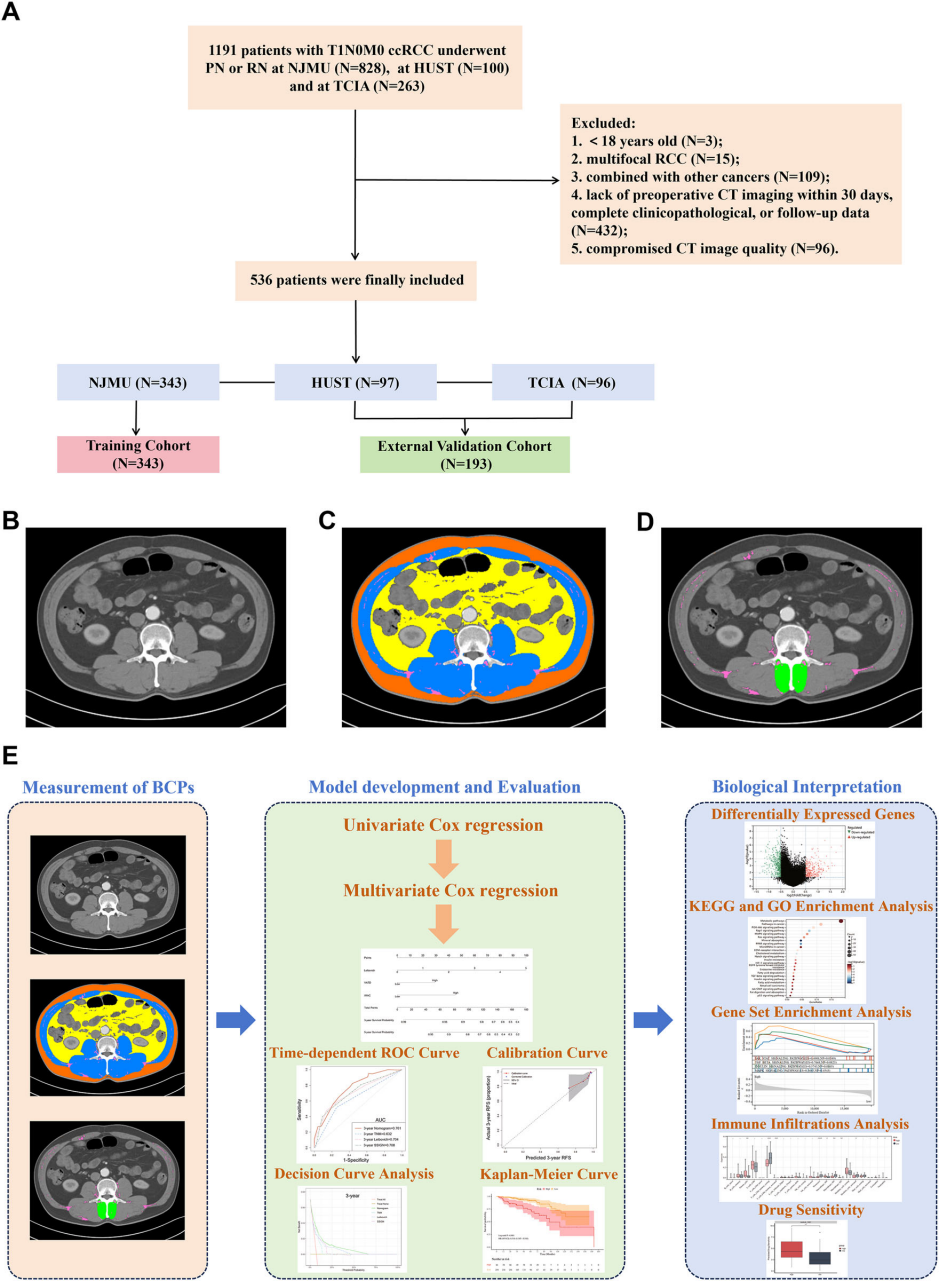
Patient selection, evaluation of body composition parameters and workflow of the research. **A** Flowchart of the patient selection. **B** Preoperative axial CT image at L3 level in a patient with T1N0M0 ccRCC. **C**, **D** Segmentation of different body compositions: blue depicts skeletal muscle, yellow depicts visceral adipose tissue, orange depicts subcutaneous adipose tissue, pink depicts intermuscular adipose tissue, and green depicts multifidus muscle. **E** Workflow of the entire research

This study retrospectively analyzed 343 patients with T1 ccRCC who underwent partial nephrectomy (PN) or radical nephrectomy (RN) at the First Affiliated Hospital of Nanjing Medical University (NJMU, a tertiary hospital located in Nanjing) between January 2009 and June 2023, serving as the training cohort for the development of predictive model. Additionally, 97 patients from Union Hospital, Tongji Medical College, Huazhong University of Science and Technology (HUST, another tertiary hospital located in Wuhan) and 96 patients from The Cancer Imaging Archive (TCIA) were included as external validation cohort. The inclusion criteria were as follows: (1) age greater than 18 years, (2) underwent CT examination within 30 days before surgery, (3) underwent PN or RN, (4) pathologically confirmed as T1N0M0 ccRCC, and (5) complete pathological and prognostic information. Exclusion criteria included: (1) multifocal RCC, (2) compromised CT image quality, (3) lack of preoperative CT imaging within 30 days, complete clinicopathological, or follow-up data, and (4) combined with other cancers. The flowchart of patient selection is shown in Fig. 1A. The primary endpoint of this study was defined as recurrence-free survival (RFS), which was calculated as the time from initial surgery to the first recurrence of the original cancer, or the date of last follow-up. Recurrence included the development of either local recurrence or distant metastasis following the operation of ccRCC. The detailed components and scoring criteria of SSIGN and Leibovich scores are shown in Table S1.

The study adhered to the latest Declaration of Helsinki ethical standards and received ethical approval from the Research Ethics Committees (NJMU Approval No. 2024-SR-386 and HUST Approval No. S187), and was conducted following the Transparent Reporting of a Multivariable Prediction Model for Individual Prognosis or Diagnosis (TRIPOD) guidelines [12] for the development and validation of prediction models.

### CT scan protocols and measurement of BCPs

BCP analysis utilized contrast-enhanced CT images acquired in the arterial phase within 30 days preoperatively. Detailed CT scanner information is provided in Table S2 across participating centers. Single-slice CT images of the third lumbar vertebra (L3) were identified and segmented, as the cross-sectional area of muscle and adipose tissue at this level correlates most strongly with the whole body composition [13, 14]. A semi-automated software (SliceOmatic version 5.0, Tomovision) was used to quantify the area and average radiodensity of various body compositions at the L3 level (Fig. 1B–D). Two trained readers who reviewed the imaging data were blinded to the clinicopathological characteristics and survival outcomes of the patients. Measured parameters included: subcutaneous adipose tissue area (SATA) and radiodensity (SATD), skeletal muscle area (SMA) and radiodensity (SMD), visceral adipose tissue area (VATA) and radiodensity (VATD), and intermuscular adipose tissue area (IMATA) and radiodensity (IMATD). The Hounsfield unit (HU) thresholds of different regions were as follows [9]: −190 to −50 HU for SAT; −150 to −30 HU for VAT and IMAT; and −29 to +150 HU for SM. Additionally, the VATA-to-SATA ratio (VSR) was calculated. The quality of SM was assessed using the intramuscular adipose tissue content (IMAC), which is the ratio of multifidus muscle radiodensity to SATD. The receiver operating characteristic (ROC) curve analysis using the SurvivalROC package was performed to determine the optimal cutoff values of each parameter separately for males and females (Table S3), due to significant gender differences in body composition [15]. For example, the optimal cutoff value for VATD was −96.47 HU in male and −94.97 HU in female; for IMAC, it was −0.51 in male and −0.36 in female. Based on the optimal cutoff values, patients were categorized into two groups.

### Development of the nomogram

No missing values were observed in any of the included variables. Univariate Cox regression analysis was conducted to identify variables with a *p*-value < 0.05 in the training cohort. These variables were then included in a multivariate Cox regression to determine independent risk factors for the development of a nomogram to predict RFS. Moreover, multicollinearity was assessed using the variance inflation factor (VIF), with a value < 2.5 indicating no significant collinearity.

### Biological interpretation and drug sensitivity analysis

Differentially expressed genes (DEGs) between high- and low-risk groups were identified using the limma package, with the filtering criteria of |log_2_-fold change (FC)| ≥ 0.5 and *p*-value < 0.05. KEGG and GO enrichment analysis were performed using the clusterProfiler package. Gene set enrichment analysis (GSEA) was carried out based on the GSEA software (http://software.broadinstitute.org/gsea/index.jsp). The mutational profiles were analyzed using the maftools package. The prediction of drug sensitivity was conducted using the oncoPredict package, which was employed to calculate the half-maximal inhibitory concentration values of the drugs. Based on DEGs, the CIBERSORT algorithm, using the IOBR package, was employed to analyze the immune infiltration levels of cells in the tumor microenvironment (TME). The overall study workflow is shown in Fig. 1E.

### Statistical analysis

The statistical analysis was performed using the R software (v.4.4.1). The categorical variables were expressed as percentages and compared using Chi-square or Fisher’s exact test. Skewed distribution variables were presented as medians (IQR), and intergroup comparisons were performed using the Mann–Whitney U test. The inter- and intra-observer variability for CT segmentation was assessed using intraclass correlation coefficients (ICC). Additionally, BCPs were analyzed as continuous variables using restricted cubic splines (RCS) regression. The discrimination ability of the nomogram was assessed using the area under the curves (AUCs) of time-dependent ROC curves and the concordance index (C-index). Moreover, model stability was assessed through k-fold cross-validation within the training cohort. To estimate the incremental predictive power, the net reclassification improvement (NRI) was calculated. The calibration curve was used to compare the predicted survival rates and the actual survival rates based on the nomogram. To minimize the overfitting bias, the nomogram underwent 1000 bootstrap resamples. Decision curve analysis (DCA) was employed to evaluate the clinical utility of the nomogram. Finally, the nomogram scores for all patients were calculated, and based on the optimal cutoff score in the training cohort, patients were classified into high- and low-risk groups. The RFS of patients in different risk groups was analyzed applying the Kaplan–Meier (KM) survival curves and compared using the log-rank test. A two-sided *p*-value < 0.05 was considered statistically significant.

## Results

### Patient characteristics

536 patients were included in this study, comprising 385 males (71.8%) and 151 females (28.2%). Among them, 174 (32.5%) patients were aged ≥ 65 years, while 362 (67.5%) patients were younger than 65 years. In the training and external validation cohorts, 53 (15.5%) and 18 (9.3%) patients, respectively, experienced postoperative tumor recurrence. In the NJMU and HUST cohorts, ipsilateral kidney recurrence was more frequent than contralateral recurrence (*n* = 19 vs. 3). The most common sites of distant metastases were the lung (*n* = 17) and bone (*n* = 10), followed by soft tissue (*n* = 5), liver (*n* = 1), adrenal gland (*n* = 1), and brain (*n* = 1). Multisite metastases (≥ 2 sites) were observed in 14 cases. The median follow-up time was 47.7 (IQR: 32.1–72.1) months for the training cohort, and 43.5 (IQR: 25.8–64.9) months for the external validation cohort. The median Leibovich score was 1 (IQR: 0–2) in both cohorts. The detailed clinicopathological characteristics and BCPs were provided in Tables 1 and S4. Inter- and intra-observer agreement for BCPs measurements demonstrated excellent reproducibility, with ICCs ranging from 0.918 (95% CI: 0.903–0.930) to 1.000 (95% CI: 0.999–1.000) (Table S5).

**Table 1:** The clinicopathological characteristics and BCPs of the patients

Variables	Training cohort (*N* = 343)	External validation cohort (*N* = 193)	*p*-value
Age, *n* (%)			0.277
< 65 years	226 (65.9)	136 (70.5)	
≥ 5 years	117 (34.1)	57 (29.5)	
Gender, *n* (%)			**0.033**
Male	257 (74.9)	128 (66.3)	
Female	86 (25.1)	65 (33.7)	
Tumor size, *n* (%)			0.076
≤ 4 cm (pT1a)	249 (72.6)	126 (65.3)	
> 4 cm (pT1b)	94 (27.4)	67 (34.7)	
Surgical procedure, *n* (%)			**< 0.001**
PN	287 (83.7)	136 (70.5)	
RN	56 (16.3)	57 (29.5)	
Laterality, *n* (%)			0.491
Left	167 (48.7)	88 (45.6)	
Right	176 (51.3)	105 (54.4)	
Nuclear grade, *n* (%)			0.804
I–II	181 (52.8)	104 (53.9)	
III–IV	162 (47.2)	89 (46.1)	
Tumor necrosis, *n* (%)			**0.018**
No	332 (96.8)	178 (92.2)	
Yes	11 (3.2)	15 (7.8)	
Leibovich score (median (IQR))	1 (0–2)	1 (0–2)	0.164
Recurrence, *n* (%)			**0.045**
Yes	53 (15.5)	18 (9.3)	
No	290 (84.5)	175 (90.7)	
VATD, *n* (%)			**0.005**
Low	203 (59.2)	90 (46.6)	
High	140 (40.8)	103 (53.4)	
IMAC, *n* (%)			0.338
Low	78 (22.7)	51 (26.4)	
High	265 (77.3)	142 (73.6)	

Bold values indicate statistical significance *p* < 0.05.

### Identification of independent prognostic factors for RFS

Univariate Cox regression analysis identified tumor size, VATD, IMAC, and Leibovich score as significant factors for RFS (all *p* < 0.05; Table 2). In the multivariate Cox regression analysis, we identified that VATD (HR = 2.34, 95% CI: 1.33–4.12), IMAC (HR = 3.60, 95% CI: 1.29–10.07) and Leibovich score (HR = 2.18, 95% CI: 1.44–3.31) were independent prognostic factors for RFS (all *p* < 0.05; Table 2). Meanwhile, no multicollinearity problems were detected among these predictors (all VIFs < 2.5; Table S6). RCS analysis confirmed that both VATD and IMAC remain independent adverse prognostic factors for RFS after adjusting for covariates, with significant nonlinear relationships (Fig. S1; *p* for overall < 0.001; *p* for nonlinear < 0.001), supporting their robust association with patient outcomes as continuous variables.

**Table 2:** Univariable and multivariable Cox regression analysis of RFS for pT1 ccRCC in training cohort

Variables	Univariate regression		Multivariate regression	
	HR (95% CI)	*p*-value	HR (95% CI)	*p*-value
Age (≥ 65 years vs. < 65 years)	1.36 (0.78–2.36)	0.282		
Gender (female vs. male)	0.59 (0.29–1.22)	0.155		
BMI (high vs. low)	0.95 (0.49–1.86)	0.889		
Tumor size (> 4 cm vs. ≤ 4 cm)	2.87 (1.66–4.97)	**< 0.001**	0.54 (0.19–1.52)	0.244
Surgical procedure (RN vs. PN)	1.54 (0.81–2.94)	0.191		
Laterality (right vs. left)	0.76 (0.44–1.31)	0.315		
SATA (high vs. low)	0.98 (0.56–1.73)	0.955		
SATD (high vs. low)	1.11 (0.57–2.20)	0.754		
SMA (high vs. low)	0.58 (0.34–1.01)	0.056		
SMD (high vs. low)	0.58 (0.34–1.01)	0.054		
VATA (high vs. low)	1.03 (0.58–1.84)	0.922		
VATD (high vs. low)	1.79 (1.03–3.13)	**0.040**	2.34 (1.33–4.12)	**0.003**
IMATA (high vs. low)	1.35 (0.77–2.38)	0.295		
IMATD (high vs. low)	1.12 (0.55–2.32)	0.753		
IMAC (high vs. low)	3.61 (1.30–10.03)	**0.014**	3.60 (1.29–10.07)	**0.015**
VSR (high vs. low)	0.53 (0.27–1.03)	0.061		
Leibovich	1.79 (1.42–2.25)	**< 0.001**	2.18 (1.44–3.31)	**< 0.001**

Bold values indicate statistical significance *p* < 0.05.

### Development and external validation of the nomogram

**Fig. 2:**
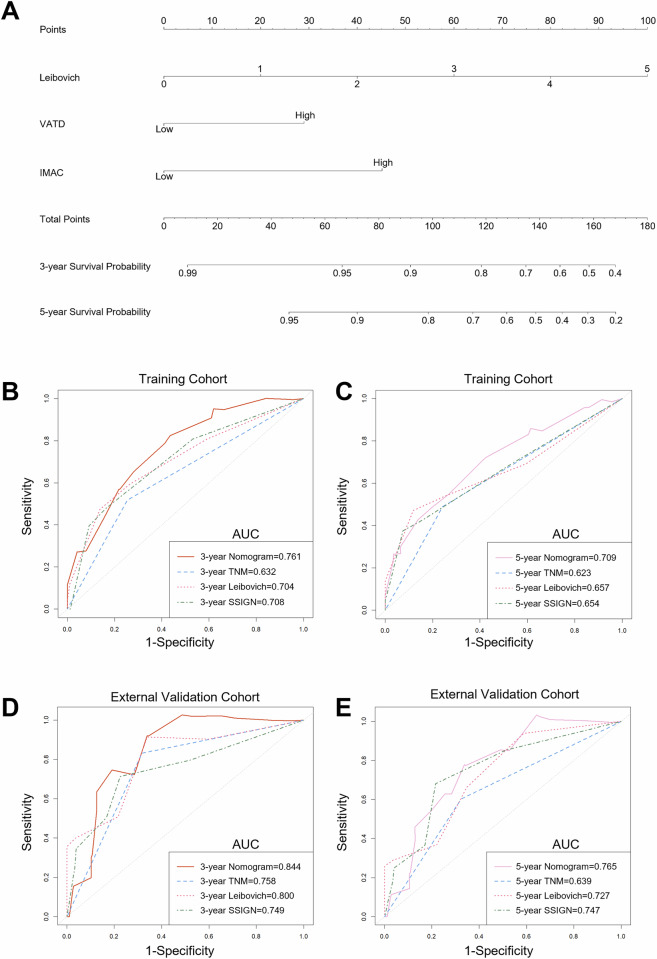
Nomogram and the receiver operating characteristic (ROC) curves. **A** Nomogram based on BCPs for predicting 3- and 5-year recurrence-free survival (RFS) (How to use: For each variable, draw a line upward to the ‘Points’ axis. Sum all partial scores and locate the total on the ‘Total Points’ axis. Draw a line downward to read the predicted survival probabilities). **B**, **C** Comparison of the ROC curves for the nomogram, TNM, Leibovich and SSIGN model in the training cohort for 3- and 5-year RFS. **D**, **E** Comparison of the ROC curves for the nomogram, TNM, Leibovich and SSIGN model in the external validation cohort for 3- and 5-year RFS

The independent prognostic factors for RFS were incorporated into a nomogram (Fig. 2A). Table S7 presents the coefficient for each variable in the nomogram, the point allocation, and predicted survival probability. The AUCs for 3- and 5-year RFS in the training cohort were 0.761 and 0.709, respectively. While in the external validation cohort, the AUCs for 3- and 5-year RFS were 0.844 and 0.765 (Fig. 2B–E). Through 200 iterations of 5-fold cross-validation within the training cohort, the model demonstrated stable predictive performance, with mean AUCs of 0.761 and 0.683 for 3- and 5-year RFS, respectively (Table S8). The C-index of the nomogram was 0.732 (95% CI: 0.655–0.810) in the training cohort and 0.766 (95% CI: 0.677–0.855) in the validation cohort (Table S9). These results outperformed the TNM stage, Leibovich and SSIGN model. To further quantify the incremental value, we applied the NRI (all NRI > 0, Table S10). In addition, a higher nomogram score was an independent predictor of poorer RFS (*p* < 0.001; Table S11) in Cox regression analyses incorporating the TNM stage, Leibovich, and SSIGN models.

The calibration curves demonstrated good consistency between the predicted and actual observation outcomes in both cohorts (Fig. S2A, B, E, F), with the corresponding slopes and intercepts detailed in Table S12. Similarly, DCA indicated that the nomogram exhibited more favorable clinical utility than other models (Fig. S2C, D, G, H). For enhanced clinical utility, we created an online nomogram (https://n0m0gram.shinyapps.io/DynNomapp/) to calculate the total score and survival probability for any given patient.

### Risk stratification of the nomogram

**Fig. 3:**
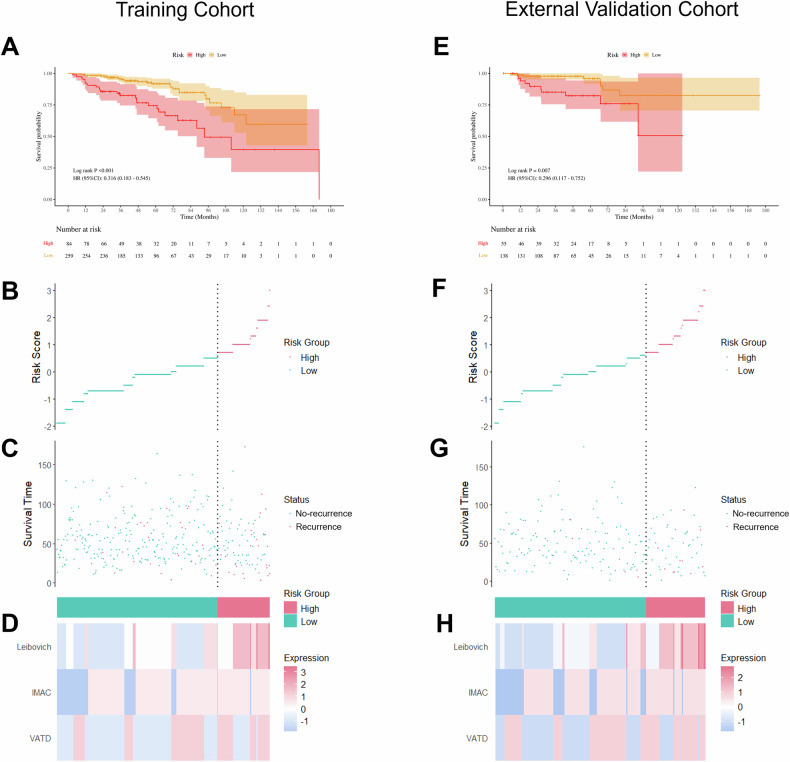
Risk stratification and distribution of risk scores in the training and external validation cohorts according to the nomogram. **A**, **E** Kaplan–Meier survival curves of RFS for patients in low- and high-risk groups. **B**, **F** Ranked dot plots showing the risk score distribution for patients in low- and high-risk groups. **C**, **G** Scatter plots showing the correlation between survival time and survival status of each patient in low- and high-risk groups. **D**, **H** Heatmaps showing the component patterns of prognostic factors in a nomogram for patients in low- and high-risk groups

The optimal cutoff nomogram score in the training cohort was 94.18, which categorized patients into low-risk (< 94.18, *n* = 259, with 27 (10.4%) recurrence events) and high-risk (≥ 94.18, *n* = 84, with 26 (31.0%) recurrence events) groups. Figure S3 shows examples of high-risk and low-risk patients. Table S13 presents the proposed clinical actions for each risk group. KM curves revealed a significant difference in RFS rates between the two risk groups (*p* < 0.001; Fig. 3A). This predefined cutoff was then directly applied to patients in the external validation cohort, where patients were classified into low-risk (*n* = 138, with 8 (5.8%) recurrence events) and high-risk (*n* = 55, with 10 (18.2%) recurrence events) groups, with a significant difference in RFS (*p* = 0.007; Fig. 3E). The risk score plots of nomogram in training and external validation cohorts indicated that as the risk score increased, the risk of recurrence increased (Fig. 3B, C, F, G). Additionally, Fig. 3D and H showed heatmaps of prognostic factors in the nomogram.

### Potential biological interpretation and drug sensitivity

**Fig. 4:**
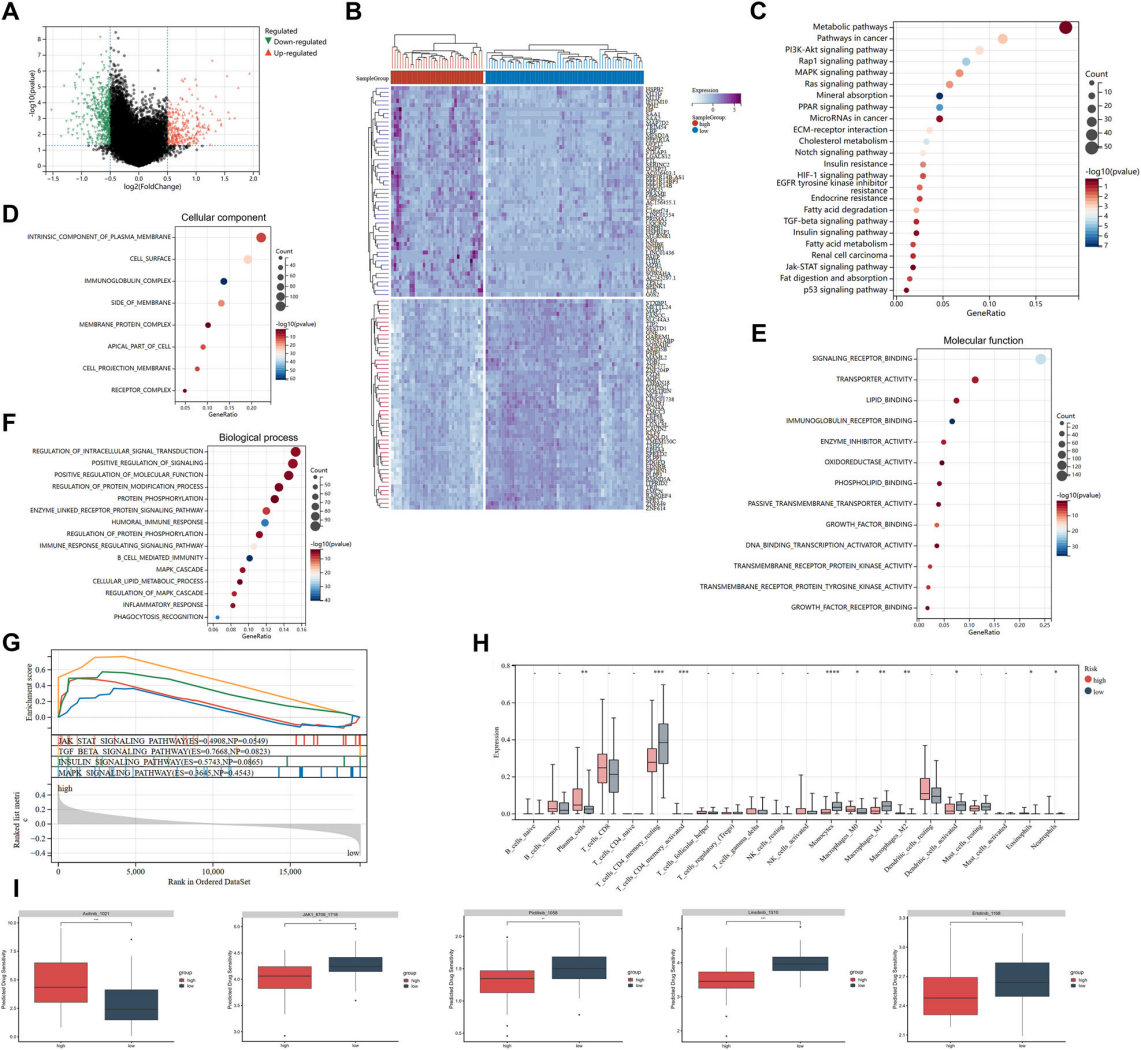
The relationship between the nomogram and genomics in the TCIA cohort. **A** Volcano plot of differentially expressed genes (DEGs) between low- and high-risk groups. **B** Heat map of DEGs between low- and high-risk groups. **C** KEGG pathways analysis of DEGs. **D**–**F** GO enrichment analysis of DEGs, including cellular component, molecular function and biological process. **G** Gene set enrichment analysis (GSEA) showing the representative pathways enriched in the high-risk group. **H** Boxplot showing the immune cell infiltration between low- and high-risk groups. **I** Boxplot showing the drug sensitivity between low- and high-risk groups

To explore potential biological mechanisms, the TCIA cohort was stratified into high- and low-risk groups by the nomogram. Transcriptomic comparison revealed 770 DEGs, with 291 genes upregulated and 479 genes downregulated in the high-risk cohort (Fig. 4A, B).

Subsequently, KEGG enrichment analysis revealed significant enrichment of DEGs in cancer-associated and metabolic signaling pathways (Fig. 4C). Furthermore, GO enrichment analysis indicated that the DEGs were predominantly enriched in cell surface (Fig. 4D), where they primarily exhibited molecular functions such as signaling receptor binding and lipid binding (Fig. 4E). These DEGs were also involved in biological processes such as inflammatory response and signal transduction (Fig. 4F). Additionally, GSEA revealed significant enrichment of the JAK-STAT, TGF-β, MAPK, and insulin signaling pathways in the high-risk group (Fig. 4G).

Immune infiltrations analysis showed 22 types of immune cell infiltrates (Fig. 4H). In high-risk patients, the infiltration ratio of M2 macrophages is higher, with a substantial depletion of memory CD4+ T cells. In contrast, in low-risk patients, the infiltration ratios of M1 macrophages, monocytes, and activated dendritic cells are higher (all *p* < 0.05).

Finally, drug sensitivity analysis revealed that high-risk patients may exhibit higher sensitivity to JAK1_8709, Pictilisib, Linsitinib, and Erlotinib, whereas low-risk patients may be more sensitive to Axitinib (Fig. 4I).

## Discussion

This multicenter study developed and validated a BCPs-based prognostic model that provided significant incremental value beyond conventional pathology-based systems, with AUC and C-index exceeding the performance of Leibovich, SSIGN, and TNM staging, highlighting the importance of systemic nutrition and metabolic status in patient prognosis. By incorporating BCPs alongside pathology, the model captures both tumor biological behavior and host metabolic status, offering a more integrated view of ccRCC pathophysiology. Furthermore, transcriptomic analysis mechanistically links elevated recurrence risk in high-risk patients to aberrant metabolic and oncogenic pathway activation, triggering TME remodeling with M2 macrophage polarization and CD4+ T-cell depletion, culminating in oncologic progression. Our drug sensitivity profiling revealed differential therapeutic options: high-risk patients demonstrated enhanced responsiveness to JAK/STAT inhibitors (JAK1_8709), PI3K inhibitors (Pictilisib), and EGFR-targeted agents (Erlotinib), whereas low-risk counterparts showed preferential sensitivity to axitinib, potentially providing valuable insights for drug development and treatment protocol selection for patients with advanced or recurrent T1 ccRCC.

The high-risk cohort is characterized by elevated VATD, IMAC, and Leibovich scores. Emerging evidence [11, 16] implicates VATD as an indicator reflecting aggressive tumor biology in ccRCC, demonstrating associations with adverse pathology and survival. Crucially, our analysis of the T1 subgroup revealed that preoperative high VATD could independently predict recurrence risk, which may be attributed to the following reasons. Elevated VATD reflects abnormal lipid consumption and metabolic dysfunction that drive chronic inflammation and hormone dysregulation [16]. Consistently, KEGG enrichment analysis and GSEA revealed significant enrichment of metabolism (e.g., fatty acid metabolism) and inflammation-related (e.g., JAK-STAT, MAPK) signaling pathways in high-risk patients. These metabolic perturbations could remodel the TME toward an inflammatory and hypoxic phenotype, manifesting as M2 macrophage accumulation and concurrent M1 macrophage depletion and thereby promoting immune suppression and tumor progression [17].

IMAC, as a novel radiomic biomarker representing intramuscular fat deposition and reduced skeletal muscle quality, is first reported in RCC. In our T1 ccRCC cohort, elevated IMAC was associated with a higher risk of recurrence. Beyond RCC, high IMAC correlates with adverse prognosis and increased postoperative complications in various malignancies [18]. Potential mechanisms involve intramuscular adipose infiltration linked to high IMAC, which induces cytokine dysregulation, promoting insulin resistance and pro-inflammatory factor secretion, thereby activating oncogenic pathways [18–20]. Our analysis of transcriptomic data also revealed this association, with DEGs in the high-risk group showing significant enrichment in insulin signaling, JAK-STAT, and MAPK signaling pathways. Furthermore, given the role of skeletal muscle as a potent immune regulator [21], increased IMAC significantly impairs immune function in tumor patients [18], correlated with reduced memory CD4+ T-cell infiltration in high-risk patients.

Clinically, the modifiable nature of preoperative BCPs adds a novel dimension to perioperative management in T1 ccRCC. Unlike immutable factors such as tumor grade or genetic alterations, skeletal muscle mass and visceral adiposity are potentially reversible through targeted interventions. For instance, preoperative nutritional optimization and structured resistance training programs have been demonstrated to enhance muscle mass and functional capacity while reducing adipose tissue accumulation in several cancer populations [22, 23], suggesting similar benefits might be extrapolated to ccRCC populations. Additionally, nutritional interventions, such as antioxidant supplements, L-carnitine, or supplements containing eicosapentaenoic acid, in combination with drugs like celecoxib and medroxyprogesterone acetate, have been demonstrated to improve skeletal muscle mass in cancer patients [24], which may be applicable to the high-IMAC population. Consequently, multimodal prehabilitation strategies integrating these nutrition, exercise, and pharmacotherapy could potentially offer survival benefits and mitigate recurrence risks in high-risk patients identified by our nomogram.

To enhance clinical applicability, we developed an online nomogram, enabling clinicians to calculate an individual patient’s total nomogram score and corresponding survival probability. To ensure reproducible radiodensity measurements across institutions, we recommend implementing a standardized CT protocol for future validation and routine application. The primary clinical utility of this tool lies in personalizing postoperative management. By contrasting with the guideline-recommended schedule for intermediate/high-risk patients (annual CT after 3 years postoperatively, then biennially after 5 years) [2], our model facilitates risk-adapted adjustments. Patients in the low-risk group may suitably follow a standardized protocol, while those identified as high-risk who demonstrate a substantially elevated recurrence probability should be recommended for intensified surveillance. This intensification could entail more frequent imaging (e.g., CT scans semiannually even after 3 years postoperatively) and may be augmented by incorporating dynamic biomarkers such as circulating tumor DNA and circulating tumor cells.

This study still has several limitations. First, its retrospective design and the lack of standardized CT protocols across centers may introduce variability in radiodensity measurements. Second, the transcriptomic and drug sensitivity analyses, reliant on public data, are exploratory and require rigorous experimental validation. Third, baseline differences between the training and external validation cohorts may introduce selection bias. Furthermore, the sex-specific BCP thresholds require validation in ethnically diverse populations to ensure generalizability of the nomogram. Therefore, future work should include prospective, multicenter studies to validate our findings, along with efforts to integrate this model into clinical decision-support systems for real-world evaluation and implementation.

## Conclusions

This study developed and externally validated a nomogram including BCPs for predicting recurrence in T1 ccRCC patients. The model demonstrated robust predictive performance, significantly outperforming existing models, and may aid clinicians in identifying high-risk patients for personalized management. Additionally, we provided a potential biological interpretation for the observed findings.

## Supplementary information

ELECTRONIC SUPPLEMENTARY MATERIAL

## Data Availability

The datasets used and/or analyzed during the current study are available from the corresponding author on reasonable request.

## Abbreviations

AUCs: Area under the curves
BCPs: Body composition parameters
ccRCC: Clear cell renal cell carcinoma
C-index: Concordance index
CT: Computed tomography
DCA: Decision curve analysis
DEGs: Differentially expressed genes
GSEA: Gene set enrichment analysis
HU: Hounsfield unit
HUST: Huazhong University of Science and Technology
ICC: Intraclass correlation coefficients
IMAC: Intramuscular adipose tissue content
IMATA: Intermuscular adipose tissue area
IMATD: Intermuscular adipose tissue radiodensity
KM: Kaplan–Meier
L3: Third lumbar vertebra
NJMU: Nanjing Medical University
NRI: Net reclassification improvement
PN: Partial nephrectomy
RCC: Renal cell carcinoma
RCS: Restricted cubic splines
RFS: Recurrence-free survival
RN: Radical nephrectomy
ROC: Receiver operating characteristic
SATA: Subcutaneous adipose tissue area
SATD: Subcutaneous adipose tissue radiodensity
SMA: Skeletal muscle area
SMD: Skeletal muscle area radiodensity
SSIGN: Stage, size, grade and necrosis
TCIA: The Cancer Imaging Archive
TME: Tumor microenvironment
TNM: Tumor-node-metastasis
TRIPOD: Transparent Reporting of a Multivariable Prediction Model for Individual Prognosis or Diagnosis
VATA: Visceral adipose tissue area
VATD: Visceral adipose tissue radiodensity
VIF: Variance inflation factor
VSR: VATA-to-SATA ratio
