# Supplementary material for: A nomogram including body composition parameters for predicting recurrence of pT1 clear cell renal cell carcinoma: a multicenter retrospective study
Source: Insights Imaging. 2026 Feb 2;17:30. doi: 10.1186/s13244-025-02202-3 (PMC12864625; doi:10.1186/s13244-025-02202-3)

A Nomogram Including Body Composition Parameters for Predicting Recurrence of pT1 Clear Cell Renal Cell Carcinoma: A Multicenter Retrospective Study

ELECTRONIC SUPPLEMENTARY MATERIAL

Table S1. The detailed components and scoring criteria of SSIGN and Leibovich scores.

SSIGN		Leibovich	
Feature	Score	Feature	Score
T stage		Primary tumor status (pathologic T	
pT1	0	pT1a	0
pT2	1	pT1b	2
pT3a	2	pT2	3
pT3b	2	pT3a	4
pT3c	2	pT3b	4
pT4	0	pT3c	4
N stage		pT4	4
pNx	0	Regional lymph node status (N stage)	
pN0	0	pNx	0
pN1	2	pN0	0
pN2	2	pN1	2
M stage		pN2	2
pM0	0	Tumor size (cm)	
pM1	4	＜10	0
Tumor size (cm)		≥10	1
＜5	0	Nuclear grade	
≥5	2	1	0
Nuclear grade		2	0
1	0	3	1
2	0	4	3
3	1	Histologic tumor necrosis	
4	3	No	0
Necrosis		Yes	1
Absent	0		
Present	2		

Table S2. The detail CT scan protocols involved in this study.

	NJMU cohort	HUST cohort
CT scanners	Siemens, Philips, and GE	Siemens, Philips, Toshiba, and GE
Slice interval, (mm)	1-1.5	1-1.5
Slice thickness, (mm)	1.5-5	0.625-5
Detector rows	64, 128, 256-section	64, 128, 256-section
Tube current	Automatic tube-current	Automatic tube-current
Tube voltage, (kV)	70-140	70-140
Contrast agent concentration, (mgI/mL)	300-350	300-350
Contrast agent dosage, (ml/kg)	1.5	1.5
Contrast agent infused rate, (ml/s)	2-3	2-3
Arterial phase scan	10s after aorta reached trigger 100Hu	10s after aorta reached trigger 100Hu

Table S3. Cut-off values of BCPs for males and females.

Variables	Male	Female
BMI, (kg/m^2)	28.83	23.47
SATA, (cm^2)	155.40	91.48
SATD, (HU)	-101.95	-100.30
SMA, (cm^2)	153.65	85.59
SMD, (HU)	39.20	30.21
VATA, (cm^2)	204.95	124.00
VATD, (HU)	-96.47	-94.97
IMATA, (cm^2)	7.66	10.35
IMATD, (HU)	-61.66	-57.81
IMAC	-0.51	-0.36
VSR	1.67	0.58

Table S4. The supplementary BCPs of the patients.

Variables	Training Cohort	External Validation cohort	*P*-Value
SATA, n(%)			＜**0.001**
Low	216 (63.0)	71 (36.8)	
High	127 (37.0)	122 (63.2)	
SATD, n(%)			0.308
Low	116 (33.8)	57 (29.5)	
High	227 (66.2)	136 (70.5)	
SMA, n(%)			＜**0.001**
Low	157 (45.8)	46 (23.8)	
High	186 (54.2)	147 (76.2)	
SMD, n(%)			0.181
Low	117 (34.1)	55 (28.5)	
High	226 (65.9)	138 (71.5)	
VATA, n(%)			**0.002**
Low	233 (67.9)	105 (54.4)	
High	110 (32.1)	88 (45.6)	
IMATA, n(%)			0.081
Low	160 (46.6)	75 (38.9)	
High	183 (53.4)	118 (61.1)	
IMATD, n(%)			0.347
Low	85 (24.8)	55 (28.5)	
High	258 (75.2)	138 (71.5)	
VSR, n(%)			0.888
Low	226 (65.9)	126 (65.3)	
High	117 (34.1)	67 (34.7)	

Table S5. Analysis of inter- or intra-observer variability in BCPs.

Variables	Inter-observer Variability		Intra-observer Variability	
	ICC	95%CI	ICC	95%CI
SATA	0.999	0.999-1.000	0.999	0.999-1.000
SATD	0.965	0.958-0.970	0.965	0.959-0.971
SMA	0.996	0.995-0.997	0.997	0.996-0.997
SMD	0.938	0.927-0.948	0.941	0.930-0.950
VATA	1.000	0.999-1.000	1.000	0.999-1.000
VATD	0.963	0.956-0.968	0.963	0.956-0.969
IMATA	0.918	0.903-0.930	0.934	0.921-0.945
IMATD	0.966	0.959-0.971	0.968	0.962-0.973
Multifidus muscle radiodensity	0.969	0.964-0.974	0.972	0.966-0.976

ICC: Intraclass correlation coefficients.

Table S6. Variance inflation factor (VIF) of variables in nomogram.

Variable	Leibovich	VATD	IMAC
VIF	1.010765	1.010211	1.013591

Table S7. Coefficients for each variable in the nomogram, point allocation, and predicted survival probability.

Predictor	Coefficient (β)	Points assigned
Leibovich score (per 1-point increase)	0.5664	20
VATD		
Low	Reference	0
High	0.8221	29.088
IMAC		
Low	Reference	0
High	1.2789	45.236
**Nomogram score**	**Estimated 3-year RFS probability**	**Estimated 5-year RFS probability**
＜94.18 (Low-risk)	＞89%	＞82%
≥94.18 (High-risk)	≤89%	≤82%

Table S8. Results of k-Fold cross-validation on the training cohort.

Parameters	Protocol	Nomogram_AUC
3-year RFS	Without cross-validation	0.761
5-year RFS	Without cross-validation	0.709
3-year RFS	5-fold cross-validation with 200 iterations	0.761*
5-year RFS	5-fold cross-validation with 200 iterations	0.683*

*Results reported as the mean.

Table S9. The C-index of the nomogram and traditional prognostic models.

Models	Training Cohort			External Validation Cohort		
	C-index	95%CI	vs. Nomogram (*P*-value)	C-index	95%CI	vs. Nomogram (*P*-value)
Nomogram	0.732[1]	0.655-0.810	-	0.766[2]	0.677-0.855	-
TNM	0.635	0.558-0.712	**0.006**	0.671	0.551-0.791	＜**0.001**
Leibovich	0.682	0.593-0.771	**0.015**	0.723	0.600-0.846	**0.026**
SSIGN	0.679	0.596-0.762	**0.019**	0.715	0.569-0.861	0.053

Table S10. NRI values of prognostic models.

Models	Training Cohort		External Validation Cohort	
	3-year NRI	5-year NRI	3-year NRI	5-year NRI
Nomogram vs. Leibovich	0.266	0.151	0.148	0.095
Nomogram vs. SSIGN	0.228	0.103	0.216	0.148
Nomogram vs. TNM	0.406	0.294	0.210	0.188

Table S11. Univariable and multivariable cox regression analysis of prognostic models.

Variables	Univariate Regression		Multivariate Regression	
	HR (95%CI)	*P*-Value	HR (95%CI)	*P*-Value
TNM Stage (T1bN0M0 vs. T1aN0M0)	2.92 (1.83-4.67)	**<0.001**	0.82 (0.32-2.11)	0.681
Leibovich	1.71 (1.42-2.05)	**<0.001**	1.03 (0.58-1.83)	0.920
SSIGN	1.61 (1.37-1.89)	**<0.001**	1.16 (0.83-1.60)	0.385
Nomogram	1.02 (1.02-1.03)	**<0.001**	1.02 (1.01-1.03)	**<0.001**

Table S12. Slopes and intercepts of the calibration curves.

Cohorts	3-year		5-year	
	Slope	Intercept	Slope	Intercept
Training cohort	0.863	0.125	0.832	0.150
External validation cohort	1.412	-0.387	0.779	0.204

Table S13. Decision-impact table.

Risk group	Estimated 3-year RFS probability [median (IQR)]	Estimated 5-year RFS probability [median (IQR)]	Proposed clinical action
Low	0.95 (0.94, 0.97)	0.92 (0.89, 0.95)	Standard follow-up
High	0.86 (0.70, 0.88)	0.76 (0.54, 0.79)	Intensified and extended surveillance/ Surveillance utilizing circulating tumor DNA and circulating tumor cells

Figure S1. Restricted cubic spline (RCS) regression analysis was performed to assess the association of VATD and IMAC with the risk of recurrence. All BCPs, tumor size, and age were included in the model as continuous variables. (**A**). The model adjusted for factors including age, gender, BMI, tumor size, surgical procedure, laterality, SATA, SATD, SMA, SMD, VATA, IMATA, IMATD, IMAC, VSR and Leibovich score. (**B**). The model adjusted for factors including age, gender, BMI, tumor size, surgical procedure, laterality, SATA, SATD, SMA, SMD, VATA, VATD, IMATA, IMATD, VSR and Leibovich score.

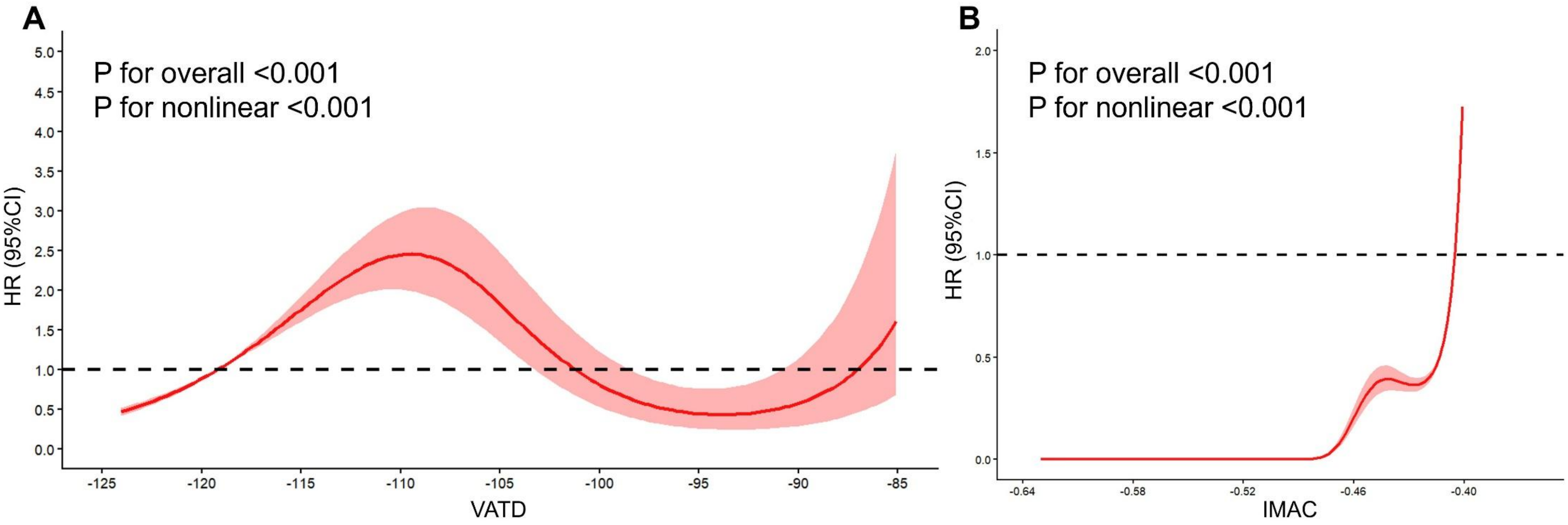

Figure S2. Calibration curves and decision curve analysis (DCA) in the training and external validation cohorts. (**A-B**). Calibration curves of the nomogram for 3- and 5-year RFS in the training cohort. (**C-D**). DCA of the models for 3- and 5-year RFS in the training cohort. When the risk threshold was between 2% and 66%, the nomogram demonstrated a net benefit that surpassed all or none strategies in predicting 3-year RFS. Besides, a similar conclusion was reached for predicting 5-year RFS when the risk threshold ranged from 5% to 87%. (**E-F**). Calibration curves for 3- and 5-year RFS in the external validation cohort. (**G-H**). DCA of the models for 3- and 5-year RFS in the external validation cohort. When the risk threshold was between 2% and 62%, there will be more net benefit than all or none strategies in predicting 3-year RFS, and when the risk threshold was between 4% and 83%, a similar conclusion was observed for predicting 5-year RFS.

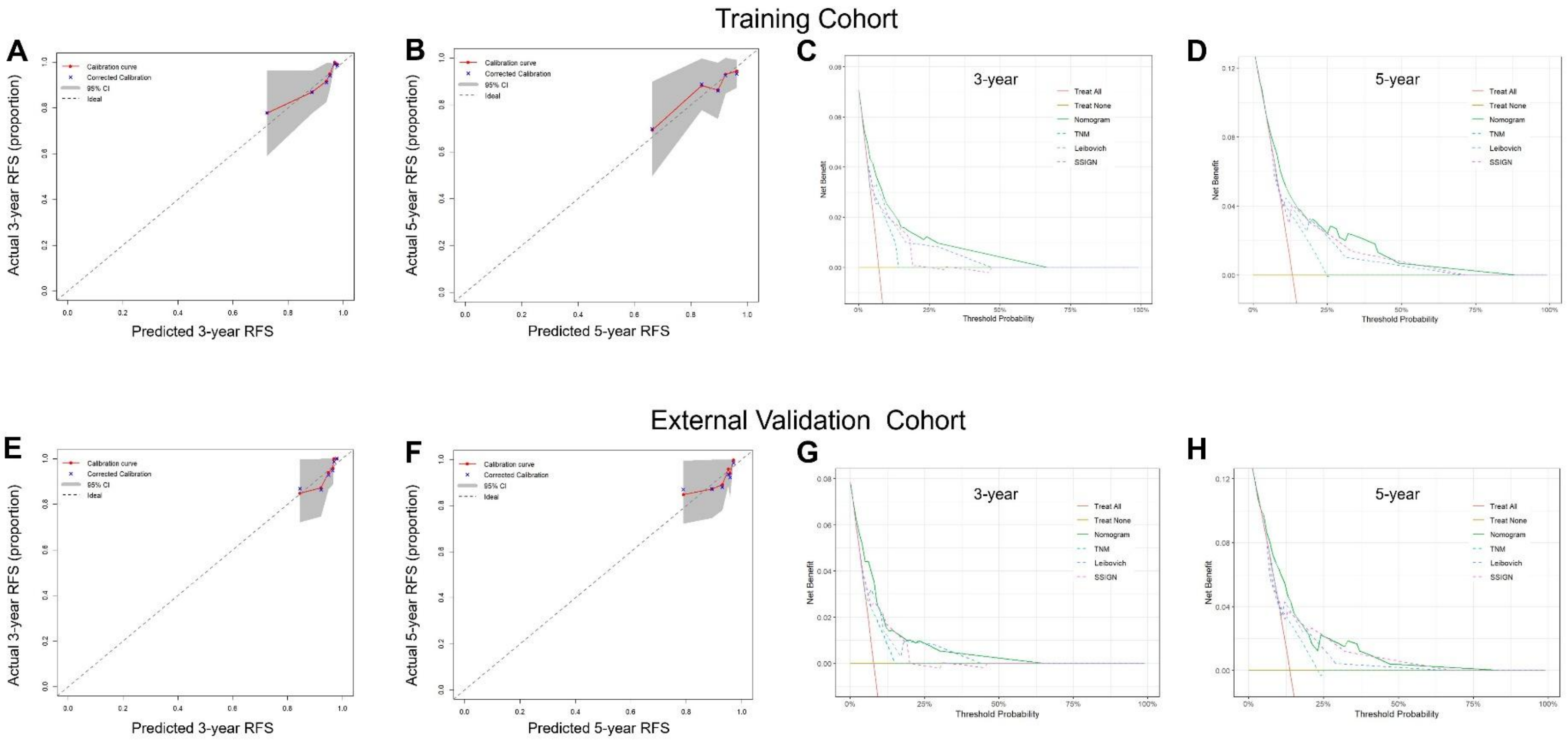

Figure S3. Examples of high-risk and low-risk patients. (**A**). A male patient with a Leibovich score of 2, a low VATD of -98.68 HU, and a high IMAC of -0.43. Using the nomogram, his total points would be: 85.154246, which places the patient in the low-risk group. This score corresponds to an approximate 3-year RFS probability of 92% and a 5-year RFS probability of 86%. At 65.5 months of postoperative follow-up, the patient remained free of tumor recurrence or metastasis. (**B**). A male patient with a Leibovich score of 2, a high VATD of -88.07 HU, and a high IMAC of -0.48. Using the nomogram, his total points would be: 114.181248, which places the patient in the high-risk group. This score corresponds to an approximate 3-year RFS probability of 82% and a 5-year RFS probability of 71%. Bone metastasis occurred at 66.4 months during postoperative follow-up.

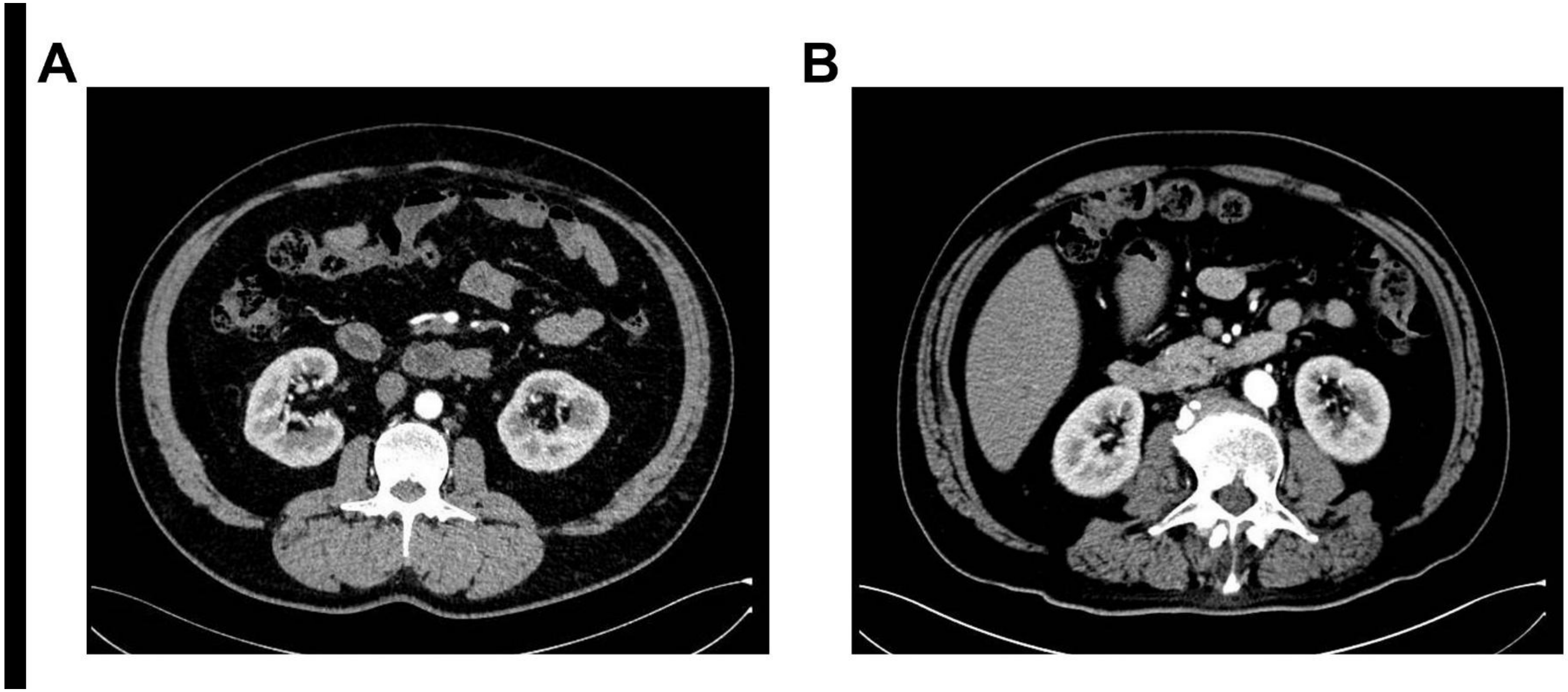

Supplement: Supplementary file 1 — ELECTRONIC SUPPLEMENTARY MATERIAL [file 13244_2025_2202_MOESM1_ESM.pdf]
